# Gibberellin-Treated Seedless Cultivation Alters Berry Fracture Behavior, Cell Size and Cell Wall Components in the Interspecific Hybrid Table Grape (*Vitis labruscana* × *Vitis vinifera*) ‘Shine Muscat’

**DOI:** 10.3390/plants15020287

**Published:** 2026-01-17

**Authors:** Hikaru Ishikawa, Kaho Masuda, Tomoki Shibuya

**Affiliations:** 1 The United Graduate School of Agricultural Sciences-Iwate University, Iwate 020-0066, Japan; 2 Horticultural Research Institute, Yamagata Integrated Agricultural Research Center, Yamagata 990-2372, Japan; 3 Faculty of Agriculture Yamagata University, Yamagata 997-8555, Japan

**Keywords:** table grape, texture, fresh, tissue structure, pectin, plant hormone

## Abstract

Gibberellin (GA)-based seedless cultivation is widely used in the skin-edible interspecific table grape (*Vitis labruscana* × *Vitis vinifera*) ‘Shine Muscat’, yet when and how GA treatment reshapes fracture-type texture during berry development remains unclear. This study aimed to identify developmental stages and tissue/cell-wall features associated with GA-dependent differences in berry fracture behavior. We integrated intact-berry fracture testing at harvest (DAFB105), quantitative histology of pericarp/mesocarp tissues just before veraison (DAFB39) and at harvest, sequential cell-wall fractionation assays targeting pectin-rich (uronic acid) and hemicellulose/cellulose-related pools at cell division period, cell expansion period and harvest, and stage-resolved RNA-Seq across the same three developmental stages. GA-treated berries had a larger diameter and showed a higher fracture load and a lower fracture strain than non-treated berries at harvest, while toughness did not differ significantly. Histology revealed thicker pericarp tissues and lower mesocarp cell density in GA-treated berries, together with increased cell-size heterogeneity and enhanced radial cell expansion. Cell wall analyses showed stage-dependent decreases in uronic acid contents in water-, EDTA-, and Na_2_CO_3_-soluble fractions in GA-treated berries. Transcriptome profiling indicated GA-responsive expression of putative cell expansion/primary-wall remodeling genes, *EXORDIUM* and *xyloglucan endotransglucosylase*/*hydrolases*, at DAFB24 and suggested relatively enhanced ethylene-/senescence-associated transcriptional programs together with pectin-modifying related genes, *Polygaracturonase*/*pectate lyase* and *pectin methylesterase*, in non-treated mature berries. Collectively, GA treatment modifies mesocarp cellular architecture and pectin-centered wall status in a stage-dependent manner, providing a tissue- and cell wall–based framework for interpreting fracture-related texture differences under GA-based seedless cultivation in ‘Shine Muscat’.

## 1. Introduction

Gibberellin (GA)-induced seedless cultivation in grape (*Vitis* spp.) has been practiced for decades, originating from small-berry cultivars including *Vitis labruscana* ‘Delaware’ [1,2]. This technique is also widely applied to the modern premium Japanese interspecific table grape (*Vitis labruscana* × *Vitis vinifera*) ‘Shine Muscat’ [3]. The effects of GA treatment on berry texture have been suggested to vary among cultivars [4], and in ‘Shine Muscat’, GA treatment has been reported to increase flesh firmness, a key texture-related quality attribute in table grapes, plausibly through changes in pericarp/mesocarp structure and cell wall (pectin) status [5,6,7,8]. In table grapes, texture is commonly quantified by puncture- or fracture-type mechanical assays on intact berries, in which the measured response reflects combined contributions of the exocarp (skin) and the fleshy pericarp, mainly the mesocarp [6,7].

At the tissue and cellular scales, such mechanical outcomes can be linked to the mesocarp cell size distribution and cell density, as well as the balance between cell-wall deformation and loss of cell-to-cell adhesion mediated by the pectin-rich middle lamella [9,10,11]. Fruit texture changes during development and ripening are associated with remodeling of the primary wall, where cellulose microfibrils form a load-bearing framework embedded in a matrix of hemicelluloses and pectins, and with modification of the pectin-rich middle lamella that governs cell-to-cell adhesion [11,12]. Xyloglucan endotransglucosylase/hydrolases (XTHs) remodel xyloglucan–cellulose associations in primary walls and can modulate wall extensibility during growth, including fruit cell expansion [13,14,15]. Since grape mesocarp development involves extensive cell expansion, stage-dependent regulation of XTHs is a plausible molecular component linking primary-wall remodeling to changes in cell size distribution and tissue mechanics [16,17]. In parallel, the mechanics of the middle lamella and cell-to-cell adhesion are strongly influenced by homogalacturonan-rich pectins. De-methyl-esterified homogalacturonan, which is rich in galacturonic acid residues, can form Ca^2+^-mediated crosslinks that influence middle-lamella mechanics and cell-to-cell adhesion [12,18]. In this study, we focused on cell wall modification, specifically enzymatic remodeling and solubilization of mesocarp wall polysaccharides, with an emphasis on galacturonan-rich pectins and xyloglucan-related hemicelluloses. Fractionation studies of grape mesocarp cell walls across veraison have shown increased solubilization and molecular-weight shifts in pectin- and xyloglucan-rich fractions, consistent with progressive weakening of middle-lamella–mediated cell adhesion and altered flesh mechanics [19,20,21]. In such assays, uronic-acid content is widely used as a compositional proxy for galacturonan-rich pectins, and variation in uronic-acid-rich fractions has been linked to firmness differences during ripening and storage [19,22].

Collectively, previous studies suggest that GA-induced seedless cultivation can increase firmness-related mechanical indices of ‘Shine Muscat’ berries and is accompanied by changes in mesocarp cell wall composition, particularly in pectin-rich fractions [4,5]. However, integrated studies that combine mechanical testing with quantitative tissue descriptors, cell wall compositional analyses, and transcriptome profiling remain limited in ‘Shine Muscat’. In particular, it remains unclear at which developmental stages GA treatment drives divergences in mesocarp cell expansion and wall-remodeling processes relevant to primary-wall extensibility and middle–lamella–associated cell adhesion. Transcriptomic studies indicate that exogenous gibberellin triggers coordinated gene-expression programs in grapevine tissues relevant to seedlessness/parthenocarpy and early berry development [23,24]. Related transcriptome shifts have also been described under practical seedless-treatment regimes in ‘Shine Muscat’ during veraison stages to over-ripening, focusing on aroma components [3]. Nevertheless, transcriptome evidence explicitly linking GA-treated seedless cultivation to berry mechanical texture traits remains limited, particularly in studies that integrate mechanical phenotyping with quantitative histology and cell wall descriptors.

Therefore, the primary objective of this study was to determine when and how GA treatment induces changes in mesocarp cell expansion and pectin solubility throughout development, and to assess the relationship between these changes and altered fracture-type mechanical behavior at harvest. Specifically, we performed intact-berry fracture testing, quantitative histology, sequential cell-wall fractionation (pectin-rich uronic-acid fractions, hemicellulose-enriched sugar fractions, and a cellulose-enriched insoluble residue), and stage-resolved RNA-Seq across key developmental stages.

## 2. Results and Discussion

### 2.1. Fracture Properties of Berries at Harvest

The harvested grape clusters are shown in Figure 1 a day after full bloom (DAFB) 105. At harvest time, the equatorial diameter of GA-treated berries was greater than that of non-treated berries (Table 1). GA-treated ‘Shine Muscat’ berries exhibited a significantly higher fracture load and a significantly lower fracture strain than non-treated berries (Table 1). These results suggest that GA treatment enhanced resistance to fracture while reducing the deformation capacity before fracture at harvest. Toughness (strain energy density to fracture) did not differ significantly between treatments; however, its mean value was lower in GA-treated berries than in non-treated berries (Table 1). Crispness is associated with brittle failure, characterized by relatively sudden fracture with limited deformation [25]. In contrast, a jelly-like texture is soft and deforms substantially before fracture, even when the work to fracture is low [25]. The combination of higher fracture load and lower fracture strain in GA-treated berries is consistent with a shift toward a more brittle-like fracture response in intact-berry testing.

### 2.2. Histological Analysis of Berry Tissue

The structure of a grape berry is illustrated in Figure 2a. In this study, the term “pericarp” collectively refers to the exocarp, mesocarp, and endocarp (Figure 2a). The exocarp is sometimes referred to as the skin. Within the pericarp, the region inside the vascular bundles was defined as the inner wall. The region outside the vascular bundles was defined as the outer wall (Figure 2a). In addition, within the mesocarp, the region inside the vascular bundles was defined as the inner side. The region outside the vascular bundles was defined as the outer side (Figure 2a). Thus, the inner wall comprises the endocarp and the inner side of the mesocarp. In contrast, the outer wall comprises the outer side of the mesocarp and the exocarp (Figure 2a). Representative equatorial cross-sections of non-GA-treated seeded and GA-treated seedless berries at 105 days after full bloom (DAFB105) are also presented (Figure 2b,c). Inner wall sides of pericarp are shown in Figure 2b and outer wall sides of pericarp are shown in Figure 2c. The pericarp is referred to as the inner wall and outer wall on the inner and outer sides of the vascular ring, respectively [26]. It has been reported that, in mature ‘Delaware’ grapes, the inner wall, outer wall, and placental tissue occupy 59.7%, 17.4% and 9.9% of the total cross-sectional area at maturity, respectively [26]. Based on previous studies, the mesocarp constitutes the majority of both the inner and outer walls [26], suggesting that the flesh is predominantly composed of the mesocarp.

**Figure 1:**
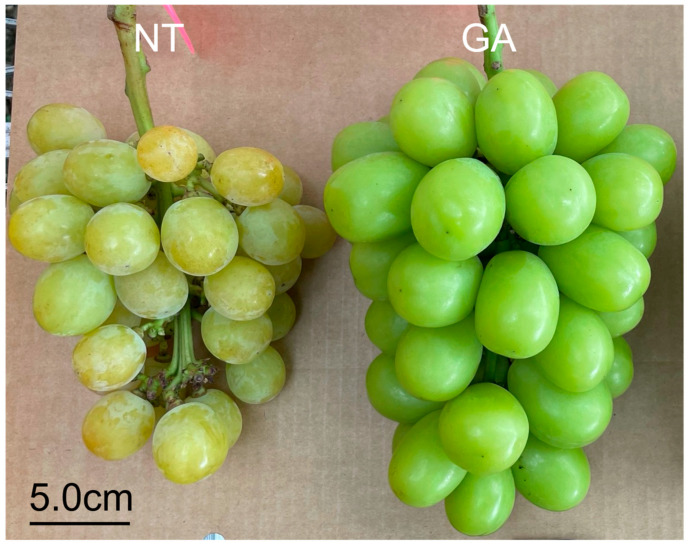
The harvested grape clusters. The photograph shows *V. labruscana* × *V. vinifera* ‘Shine Muscat’ grape clusters at DAFB105 (scale bar = 5.0 cm). GA, GA-treated clusters; NT, non-treated clusters.

**Figure 2:**
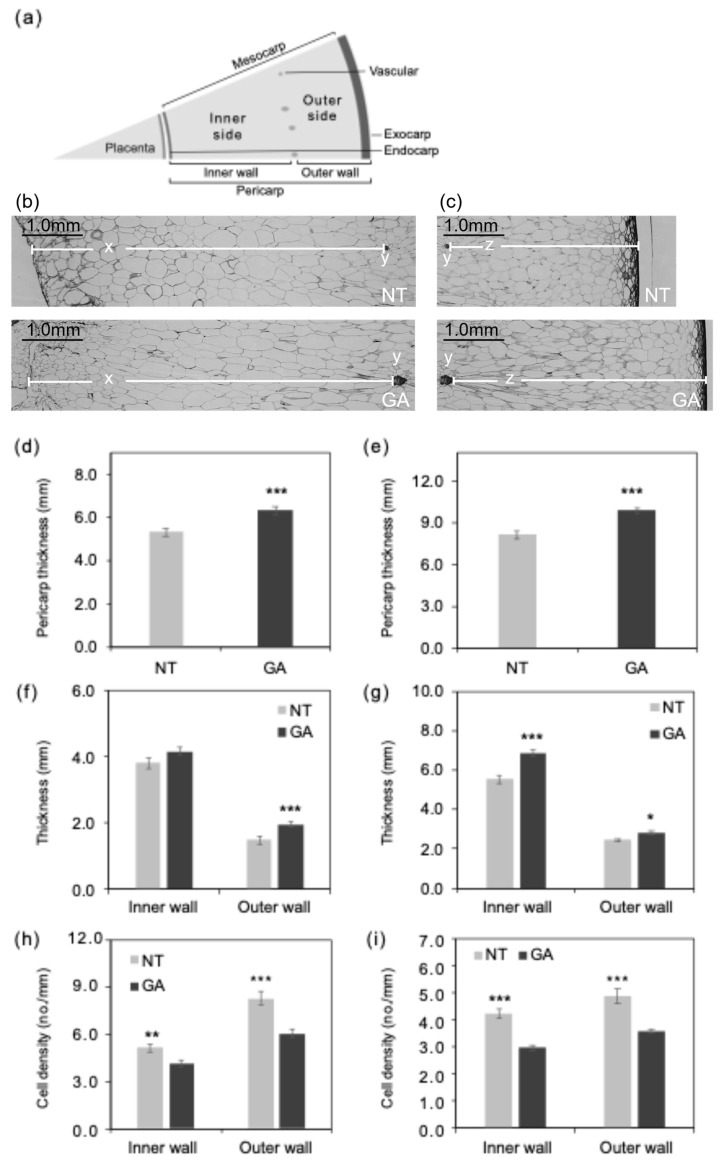
Histological characteristics of the pericarp tissue structure. (**a**) Schematic diagram of the structure of a fresh grape berry. Pericarp structure near the equatorial plane (**b**) inner wall and (**c**) outer wall side at DAFB105 (scale bars = 1.0 mm). x, inner wall of pericarp; y, vascular; z, outer wall of pericarp. (**d**,**e**) Pericarp thickness at DAFB39 and DAFB105, respectively. (**f**,**g**) Thickness of the inner and outer walls of the pericarp at DAFB 39 and DAFB 105, respectively. Inner wall, inner wall of the pericarp; Outer wall, outer wall of the pericarp. (**h**,**i**) Mesocarp cell density on the inner and outer sides at DAFB 39 and DAFB 105, respectively. Inner side, inner side of the mesocarp; Outer side, outer side of the mesocarp. *, *p* < 0.05; **, *p* < 0.01; ***, *p* < 0.001; Welch’s *t*-test; *n* = 18–23. Error bars indicate standard error. GA, GA-treated berries; NT, non-treated berries.

**Figure 3:**
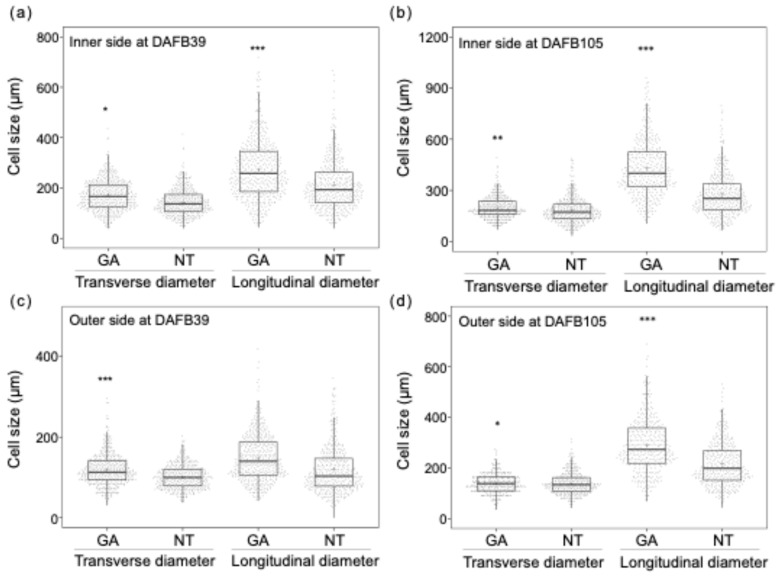
Cell size distributions of the (**a**) inner and (**b**) outer sides of the mesocarp at DAFB39 and the (**c**) inner and (**d**) outer sides of the mesocarp at DAFB105 (*, *p* < 0.05; **, *p* < 0.01; ***, *p* < 0.001; F-test for equality of variances; *n* = 360–460 cells). The box plot displays the interquartile range (IQR) with the median indicated by a horizontal line. Whiskers extend to 1.5 × IQR, jittered points represent individual cell measurements, and the mean is denoted by a plus sign (+). Inner side, inner side of the mesocarp; Outer side, outer side of the mesocarp; GA, GA-treated berries; NT, non-treated berries; Transverse, circumferential on the equatorial plane of the berry; Longitudinal, radial on the equatorial plane of the berry.

The pericarp of GA-treated berries was thicker than that of non-treated berries at DAFB39 and DAFB105 (Figure 2d,e). The outer wall of the pericarp was thicker in GA-treated berries than in non-treated berries at DAFB39 (Figure 2f). In contrast, both the inner and outer walls of the pericarp were thicker in GA-treated berries at DAFB105 (Figure 2g). The pericarp was thicker in GA-treated berries than in non-treated berries (Figure 2d,e), which was consistent with the larger berry size observed in GA-treated berries (Figure 1). Cell density in both the inner and outer sides of the mesocarp was lower in GA-treated berries than in non-treated berries at DAFB39 and DAFB105 (Figure 2h,i). The transverse (circumferential) and longitudinal (radial) diameters of cells in the inner side of the mesocarp exhibited greater variability in GA-treated berries than in non-treated berries at DAFB39 (Figure 3a), and the transverse diameter of cells in the outer side of the mesocarp also showed higher variability in GA-treated berries at this at DAFB39 (Figure 3b). Both transverse and longitudinal cell diameters in the inner and outer sides of the mesocarp displayed greater variability in GA-treated berries than in non-treated berries at DAFB105 (Figure 3c,d).

**Figure 4:**
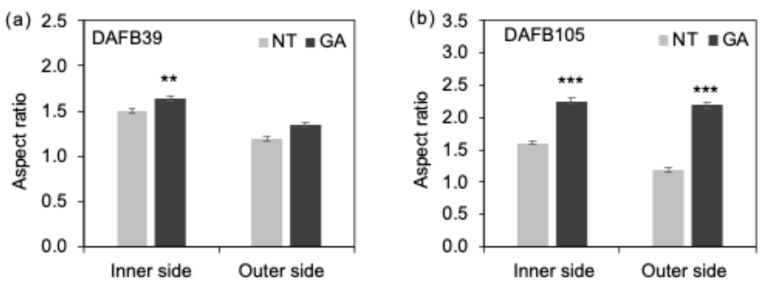
Cell shape. Cell aspect ratio at (**a**) DAFB39 and (**b**) DAFB105, respectively (**, *p* < 0.01; ***, *p* < 0.001; Welch’s *t*-test; *n* = 360–460 cells). Error bars indicate standard error. Inner side, inner side of the mesocarp; Outer side, outer side of the mesocarp. GA, GA-treated berries; NT, non-treated berries.

In addition, the longitudinal-to-transverse ratio of cells in the inner side of the mesocarp was higher in GA-treated berries at both DAFB39 and DAFB105, and a similar tendency was observed in the outer side of the mesocarp at DAFB105 (Figure 4a,b). Consistent with these results, the distributions of cell sizes in both the inner and outer sides of the mesocarp at DAFB105 were broader in GA-treated berries than in non-treated berries (Figure S1). These observations suggest that GA treatment promoted radial cell expansion in the mesocarp and increased cell size heterogeneity relative to non-treated berries.

Cell-size heterogeneity can influence tissue-scale mechanics by altering the distribution of stresses within the cellular network. In strawberry (*Fragaria* × *ananassa*), volumetric cell-size distributions were quantitatively associated with tissue elastic modulus and failure stress, with less heterogeneous distributions generally corresponding to mechanically stronger tissues [27]. Although comparable quantitative evidence remains limited in grape berries, GA_3_-induced parthenocarpy in ‘Delaware’ has been reported to modify patterns of cell proliferation and enlargement across pericarp tissues [28], supporting the view that GA treatment can shift the balance between cell division and expansion. Consistent with the idea that cellular architecture and wall chemistry jointly condition texture, firmness differences between inner and outer mesocarp tissues in table grapes have also been linked to pectin methyl-esterification status and cell-wall-associated calcium [29]. Previous studies have shown that tomato (*Solanum lycopersicum*), a model for fruit development, treated with GA_3_ alone had fewer cell layers but larger mesocarp cell diameters than pollinated fruits at 21 days after anthesis (DAA) [30], which is consistent with our results in grape. In addition, Lu et al. [31] suggested that GA stimulates mesocarp cell expansion in grape, whereas auxin and cytokinin stimulate mesocarp cell division. Therefore, the decreased cell density observed in this study is likely a secondary consequence of enhanced cell enlargement.

### 2.3. Plant Cell Wall Component Analysis

**Figure 5:**
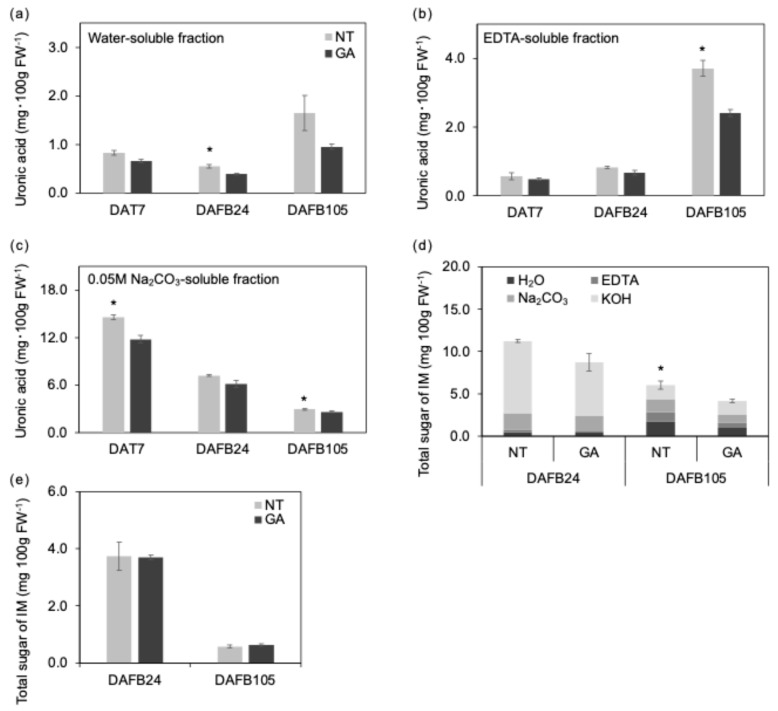
Cell wall components in the mesocarp and endocarp. (**a**) Water-soluble, (**b**) EDTA-soluble, and (**c**) 0.05 M Na_2_CO_3_-soluble uronic acid contents were pectin-related. (**d**) Hemicellulose-related sugar content. H_2_O, water-soluble fraction; EDTA, EDTA-soluble fraction; Na_2_CO_3_, 0.05 M Na_2_CO_3_-soluble fraction; KOH, 4 M KOH-soluble fraction. (**e**) Insoluble material (IM). *, *p* < 0.05; *n* = 3. Error bars indicate standard error. GA, GA-treated berries; NT, non-treated berries.

The uronic acid content in the water-soluble fraction of GA-treated berries was lower than that of non-treated berries at DAFB24 (Figure 5a). The uronic acid content in the EDTA-soluble fraction of GA-treated berries was also lower than that of non-treated berries at DAFB105 (Figure 5b). In addition, at DAFB10 and DAFB105, the uronic acid content in the Na_2_CO_3_-soluble fraction was lower in GA-treated berries than in non-treated berries (Figure 5c). The EDTA-soluble fraction is considered an operational indicator reflecting pectins that were retained in the cell wall via ionic interactions with cations, primary Ca^2+^, and subsequently solubilized by chelation [32,33]. Because the solubility of cell wall polysaccharides defines these fractions, quantitative differences should be interpreted in conjunction with qualitative changes such as molecular weight and the degree of de-methylesterification. Moreover, the relationship between pectin modification and fruit texture during ripening is not straightforward, and changes in fraction abundance alone do not necessarily explain differences in stiffness or firmness [9,10].

De-methylesterification of homogalacturonan can promote Ca^2+^-mediated crosslinking and facilitate the formation of pectin–Ca gels with an egg-box-like structure [34,35]. Thus, an increase in the EDTA-soluble fraction may contribute less to directly enhancing the maximum tissue strength than to modulating cell-to-cell adhesion and gel-like viscoelastic behavior [18,35,36]. In fruits, the status of cell-to-cell adhesion has been shown to affect fracture mode (cell separation vs. cell rupture) and associated mechanical properties [37]. Accordingly, differences in pectin status and intercellular adhesion could alter fracture mode, such that maximum strength and deformation tolerance do not necessarily change in the same direction. In this context, the lower fracture load and higher fracture strain observed in GA-non-treated berries at the mature stage may reflect reduced maximum strength due to ripening- and senescence-associated cell wall reorganization, while differences in pectin status and cellular arrangement may have relatively maintained or enhanced deformation tolerance and energy absorption. Because Honda et al. [3] showed that nontreated ‘Shine Muscat’ berries ripen later than GA-treated berries on sugar and aromatic component under a comparable plant growth regulator regime, the greater softness of our nontreated berries is unlikely to reflect over-ripening. By contrast, because no significant difference was detected in total sugar content in the 4 M KOH-soluble fraction (Figure 5d), quantitative changes in alkali-soluble polysaccharides (hemicelluloses enriched fraction) were not prominent under our analytical conditions. The whole total sugar content was higher in the non-treated fruit (Figure 5d). Likewise, the absence of a clear difference in total sugar content in the insoluble material suggests that quantitative changes in cellulose-related components (Figure 5d) were not substantial.

Overall, these results suggest that, in the mesocarp at maturity, GA treatment may have stepwise effects on the balance between pectin solubilization and retention and on pectin structural status, thereby contributing to textural differences. In our study, the lower EDTA-soluble uronic acid content in GA-treated berries at DAFB105 may reflect altered ionically bound pectin pools and/or reduced Ca^2+^-associated pectin retention, which could influence cell-to-cell adhesion and fracture behavior at maturity.

### 2.4. Transcriptome Analysis

**Figure 6:**
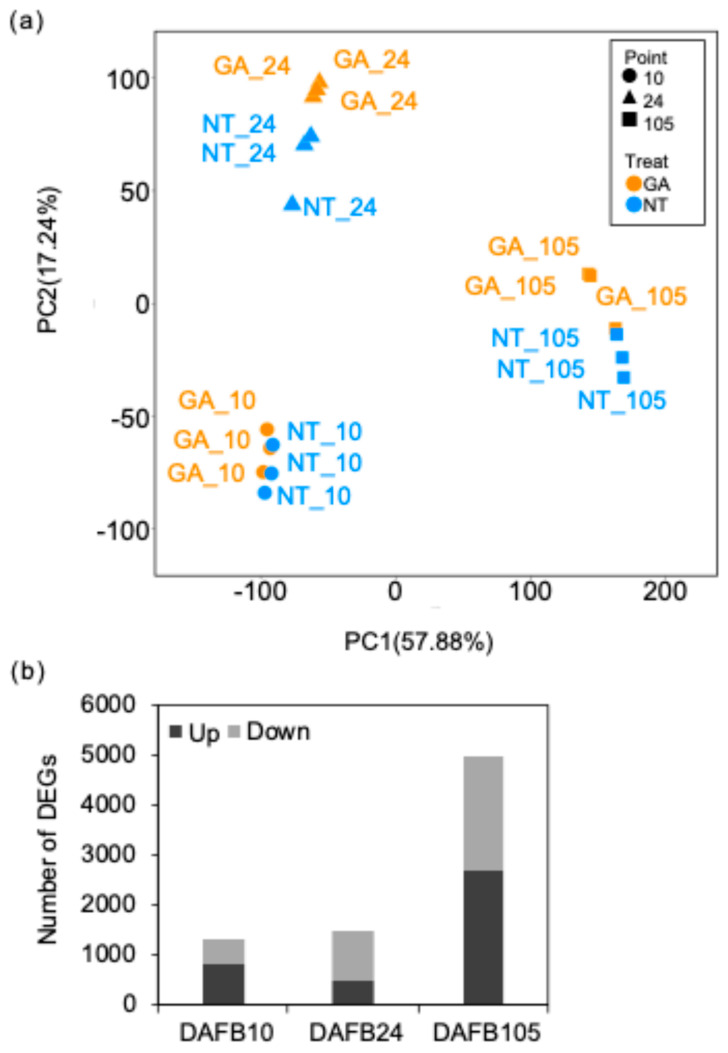
RNA-Seq summary. (**a**) PCA based on gene expression levels. The first principal component (PC1, 57.88% of the variance explained) and the second principal component (PC2, 17.24%). GA, GA-treated berries, NT, non-GA-treated berries. (**b**) Number of DEGs. DEGs were genes with a *padj* < 0.05. Up, higher expression in GA-treated berries than non-treated berries; down, lower expression in GA-treated than NT berries. 10, DAFB10, 24, DAFB24, 105, DAFB105 (harvest stage).

Approximately 20,079,000–26,106,000 reads were obtained from each sample (Table S1). Principal component analysis (PCA) showed that the first principal component (PC1; 57.88% of the variance) separated at DAFB105 from the other stages (Figure 6a). The second principal component (PC2; 17.24%) primarily reflected stage-dependent variation, with the treatment effect also partially captured along this axis (Figure 6a). Differential expression analysis (*padj* < 0.05) identified 1326 genes at DAFB10, of which 824 were upregulated and 502 were downregulated in GA-treated berries relative to non-treated berries (Figure 6b). 1475 genes were differentially expressed (494 upregulated and 981 downregulated in GA-treated berries) at DAFB24, whereas 4964 genes were differentially expressed at DAFB105 (2668 upregulated and 2296 downregulated in GA-treated berries) (Figure 6b). The differential expression genes (DEGs) between GA-treated vs. non-treated berry were showed in Tables S2–S4.

**Figure 7:**
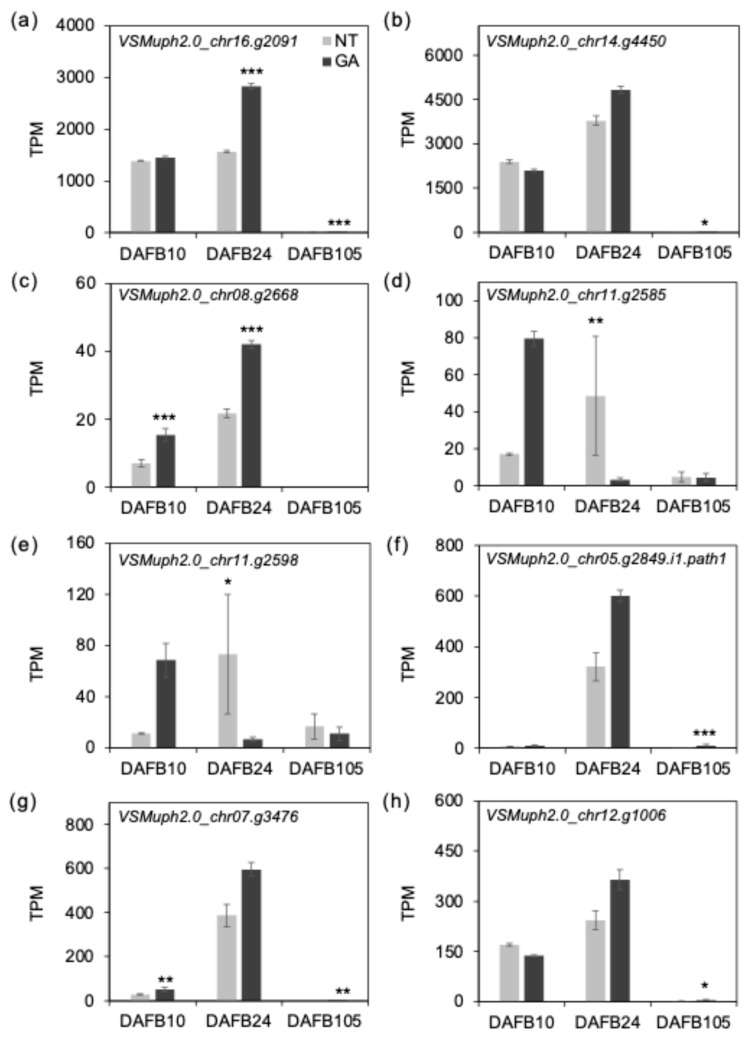
Expression of putative cell expansion-related genes. TPM values of (**a**) *VSMuph2.0_chr16.g2091* (EXORDIUM-Like 3), (**b**) *VSMuph2.0_chr14.g4450* (expansin A1), (**c**) *VSMuph2.0_chr08.g2668* (xyloglucan endotransglucosylase/hydrolase 32), (**d**) *VSMuph2.0_chr11.g2585* (xyloglucan endotransglycosylase 6), (**e**) *VSMuph2.0_chr11.g2598* (xyloglucan endotransglycosylase 6), (**f**) *VSMuph2.0_chr05.g2849* (xyloglucan endotransglucosylase/hydrolase 15), (**g**) *VSMuph2.0_chr07.g3476* (xyloglucan endotransglucosylase/hydrolase 7) and (**h**) *VSMuph2.0_chr12.g1006* (xyloglucan endotransglucosylase/hydrolase 9). Error bars indicate standard error, *n* = 3. *, *padj* < 0.05; **, *padj* < 0.01; ***, *padj* < 0.001. GA, GA-treated berries; NT, non-treated berries.

Gene expression levels were summarized as transcripts per million (TPM), a gene length- and library size-normalized measure, and TPM values were used in the following analyses and figures [38,39]. To interpret the transcriptome data in relation to our anatomical observations, we focused on genes putatively involved in cell expansion and cell wall remodeling, including EXORDIUM/EXORDIUM-like (EXO), expansins (EXP), and xyloglucan endotransglucosylase/hydrolases (XTH/XET). The expression of *VSMuph2.0_chr16.g2091*, which is predicted to encode an EXORDIUM_like protein, was higher in GA-treated berries at DAFB24 (Figure 7a), and *VSMuph2.0_chr14.g4450*, annotated as an expansin-like gene, also showed a tendency toward higher expression at DAFB24 (Figure 7b). EXO has been reported to be involved in the regulation of cell proliferation and to promote cell expansion in *Arabidopsis thaliana* [40,41], suggesting a potential role in cell enlargement in fruit tissues. Expansins are cell wall-localized proteins that promote cell expansion by inducing cell wall loosening [42,43]. Transcript levels of *VSMuph2.0_chr08.g2668*, annotated as xyloglucan endotransglucosylase/hydrolase 32-like (XTH32-like), were higher in GA-treated berries at DAFB10 and DAFB24 (Figure 7c). *VSMuph2.0_chr08.g2668* was annotated as a group IIIA XTH (*VIT_208s0007g04950*), a clade enriched for enzymes with xyloglucan endohydrolase (XEH) activity [44]. Transcripts of *XTH6-like* genes were lower in GA-treated berries at DAFB24 (Figure 7d,e). In contrast, the more highly expressed *XTH-like* genes tended to show higher transcript levels in GA-treated berries at the same stage (Figure 7f–h). Together, these patterns indicated that GA effects on the XTH family are gene-dependent and may shift the relative contribution of different XTH members during early berry development. Because several GA-upregulated *XTH-like* genes shown here are among the more abundant *XTH* transcripts examined, the aggregate *XTH* transcript level across this subset could be higher under GA treatment at DAFB24. XTHs remodel xyloglucan, a major hemicellulose associated with cellulose microfibril surfaces in primary walls; most catalyze transglycosylation reactions, whereas a subset shows higher hydrolase activity, thereby modulating microfibril–matrix coupling and primary-wall extensibility in a context-dependent manner [13,14,15]. In addition, the upregulation of putative cell expansion–related genes (including *EXO-like* transcripts) in GA-treated berries during the active cell enlargement stage, together with the GA-associated induction of several *XTH-like* genes, is consistent with a scenario in which GA enhances primary-wall extensibility through activation of xyloglucan/hemicellulose remodeling. These coordinated changes may facilitate radial mesocarp cell expansion and contribute to the increased mean cell size and broader cell-size distributions observed in GA-treated berries.

**Figure 8:**
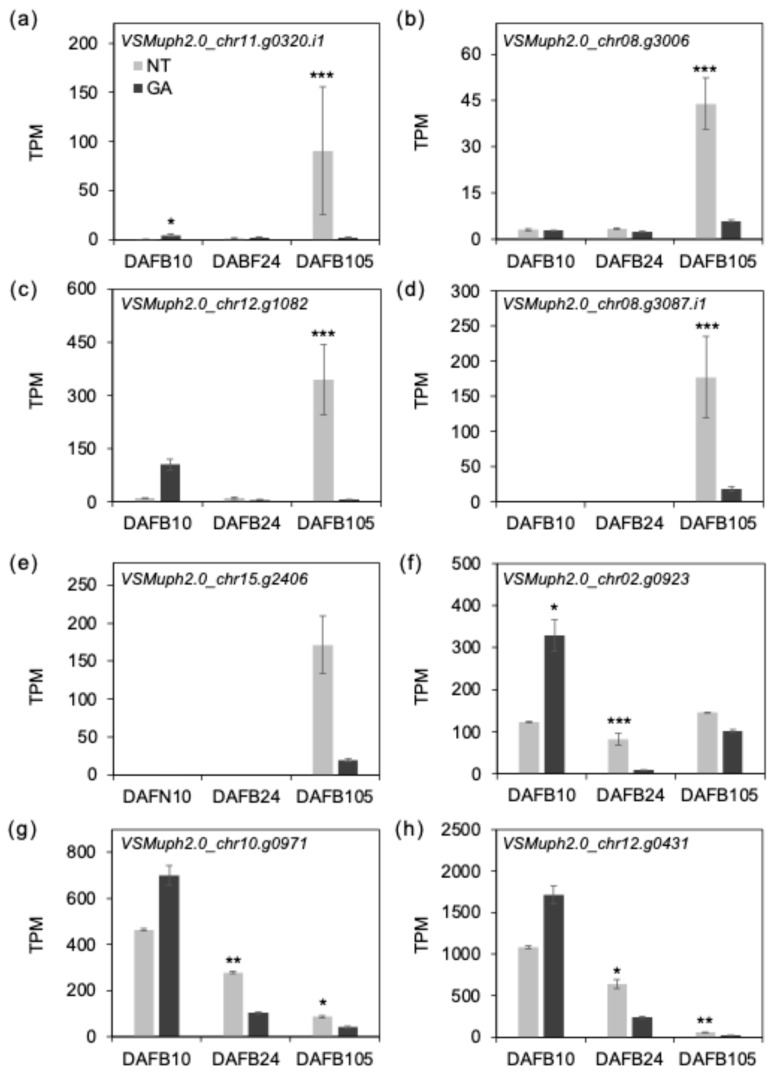
Expression of putative ethylene-related genes. TPM values of (**a**) *VSMuph2.0_chr11.g0320* (ACC oxidase 1), (**b**) *VSMuph2.0_chr08.g3006* (NAC domain containing protein 87), (**c**) *VSMuph2.0_chr12.g1082* (Wound-responsive family protein), (**d**) *VSMuph2.0_chr08.g3087.i1* (Pectin lyase-like superfamily protein), (**e**) *VSMuph2.0_chr15.g2406* (Plant invertase/pectin methylesterase inhibitor superfamily), (**f**) *VSMuph2.0_chr02.g0923* (ethylene responsive element binding factor 1), (**g**) *VSMuph2.0_chr10.g0971* (erf domain protein 9) and (**h**) *VSMuph2.0_chr12.g0431.1* (erf domain protein 9). Error bars indicate standard error, *n* = 3. *, *padj* < 0.05; **, *padj* < 0.01; ***, *padj* < 0.001. GA, GA-treated berries; NT, non-treated berries.

To examine transcriptional differences at harvest (DAFB105), we screened DEGs showing large expression changes (|log_2_FC| ≥ 2, DESeq2) and identified an *aminocyclopropane-1-carboxylate oxidase 1-like (ACO1-like)* gene (*VSMuph2.0_chr11.g0320*, Figure 8a), which exhibited a extremely high fold change (|log_2_FC| > 5, Table S4). ACC oxidase catalyzes the final step of ethylene biosynthesis [45], and ethylene-associated ripening/senescence programs can contribute to softening through regulation of pectin-related cell wall remodeling [46]. In grape, ripening/softening–associated expression of pectin-modifying genes in the skin supports active pectin remodeling during maturation [47], so we used the *ACO1-like* expression pattern to prioritize similarly regulated candidate genes. Because *ACO1-like* transcript levels showed substantial among-replicate variability, we considered that some biologically linked genes might not be recovered as DEGs under stringent multiple-testing correction; therefore, we additionally performed a co-expression-based prioritization using the *ACO1-like* expression profile. Using Pearson correlation on log2(TPM + 1) values (r ≥ 0.8), we identified 315 genes that were highly correlated with the *ACO1-like* gene (Table S5). As a result, *NAC87 like*, *wound-responsive* genes, and genes annotated polygalacturonase/pectate lyase—like and pectin methylethterase-like proteins were found to be preferentially and strongly expressed in non-treated berries at maturity (Figure 8b–e). PMEs de-methyl-esterify homogalacturonan (HG), thereby shifting the balance between Ca^2+^-mediated crosslinking and the susceptibility of HG to solubilization and depolymerization by pectin-depolymerizing enzymes such as polygalacturonases and pectate lyases [34,36,48]. *NAC87-like* is annotated as a homolog of *A. thaliana ANAC087*, which has been implicated in programmed cell death [49] and was recently shown to positively regulate age-dependent leaf senescence [50]. This behavior is consistent with the possible activation of a senescence-like/stress-associated transcriptional program in non-treated mature berries in the present study. In addition, several *ethylene responsive factor (ERF)-like* gene transcripts with relatively high abundance at maturity, including *VSMuph2.0_chr12.g0431* and *VSMuph2.0_chr10.g0971*, were significantly higher in non-treated berries (Figure 8f–h). These results support the possibility that non-treated mature berries exhibit a relatively enhanced ethylene-associated program coupled with stress-/senescence-like regulation, which may facilitate pectin remodeling and contribute to the softer and/or gummy fracture behavior observed at harvest. Notably, *ERF-like* transcripts exhibited treatment-dependent differences as early as DAFB24. While *ERF–ACO1* patterns were not tightly aligned at early stages, *ACS1* showed a broadly similar trend to the *ERF* profile at DAFB10–24 (Figure S2), suggesting that ethylene/*ERF*-associated transcriptional states may diverge early during the cell enlargement phase.

## 3. Materials and Methods

### 3.1. Plant Material

Interspecific grapevines (*Vitis labruscana* × *V. vinifera*) ‘Shine muscat’ grown in 2023 at the experimental vineyard of Horticultural Research Institute, Yamagata Integrated Agricultural Research Center, Yamagata, Japan, was used in this study. For the compositional analyses and RNA-Seq, three clusters per treatment were sampled, each taken from a different fruiting shoot within the same vine. For each cluster, ten berries were collected and pooled to generate one biological replicate (*n* = 3). Each cluster was treated as an independent biological replicate. For the fracture test, berries were sampled at 105 days after full bloom (DAFB105, harvest time), and 6–10 berries were collected from each of 5–8 clusters per treatment (*n* = 4–8). For histological analysis, berries were sampled at DAFB39 (veraison stage for seedless berries) and DAFB105 (harvest time), with 5–10 berries each collected from the three clusters per treatment. For cell wall component analysis and gene expression analysis was performed on berries sampled at DAFB10 and DAFB24, representing the early (cell proliferation–dominant) and subsequent (active cell-expansion) phases, respectively, and at DAFB105, which corresponds to the harvest stage. Ten Berries were collected from each of three clusters per treatment (*n* = 3). For these analyses, the exocarp, placenta, and seeds were removed from each berry to obtain mesocarp and endocarp tissues. The samples were immediately frozen in liquid nitrogen and stored at −80 °C until use.

### 3.2. Plant Hormone Treatment

All clusters on the experimental vines were sprayed with a 1:2000 dilution of mepiquat chloride (Nisso Flaster; Nippon Soda Co., Ltd., Tokyo, Japan) at 9 days before full bloom. In the GA-treated group, cluster dipping with 200 mg/L streptomycin (Mitsui Chemicals Crop & Life Solutions, Inc., Tokyo, Japan) was performed at 8 days before full bloom according to a conventional seedless cultivation protocol.

Gibberellin (GA) treatment was applied twice, at DAFB3 and DAFB15. For the first treatment, flower clusters were dipped in a mixed solution containing 25 mg/L GA_3_ (Sumitomo Chemical Co., Ltd. Tokyo, Japan), and 5 mg/L CPPU (Fulmet; Sumitomo Chemical Co., Ltd., Tokyo, Japan). For the second treatment, fruit clusters were dipped in a solution containing 25 mg/L GA_3_.

### 3.3. Fruit Instrumental Texture Analysis

All berries were first measured for berry equatorial largest diameter and then subjected to a fracture test using a creep meter (RE2-3305C; YAMADEN Co., Ltd., Tokyo, Japan) at the Horticultural Research Institute, Yamagata Integrated Agricultural Research Center. A cylindrical plunger (3.0 mm in diameter) was driven through the berry near the equatorial region at a table speed of 1 mm s^−1^. The amplifier gain was set to ×10. Each intact berry was positioned with the equatorial plane perpendicular to the plunger axis and compressed until the first fracture event occurred, which was identified as the first peak force followed by an abrupt force drop on the force–displacement curve. The force at the first fracture event was recorded as the fracture load (N). Fracture strain (%) was calculated as (Δ*L*1st_fracture_/*D* × 100), where Δ*L*1st_fracture_ is plunger displacement from initial contact to first fracture (mm) and *D* is equatorial berry diameter (mm). Toughness (strain energy density to first fracture; kJ m^−3^) was computed as the area under the force–displacement curve up to the first fracture event, normalized by the product of plunger cross-sectional area and equatorial berry diameter.

### 3.4. Histological Analysis

All berries used for histological observation were sliced to approximately 2.0 mm thickness around the equatorial region and fixed in 50–100 volumes of FAA solution (100% ethanol: glacial acetic acid: formalin: ultrapure water = 12:1:1:6). Samples were vacuum-infiltrated and stored in glass bottles until use [51]. Samples preserved in FAA solution were washed twice with 60% ethanol and then gradually transferred to 100% ethanol for solvent exchange [51]. During the exchange procedure, samples were vacuum-infiltrated under reduced pressure and then immersed for at least 12 h at each step [51]. For resin embedding, Technovit^®^ 7100 (Kulzer, Hanau, Germany) was used. Resin substitution solutions were prepared by mixing Technovit^®^ 7100 with hardener I (100 mL Technovit^®^ 7100 per 1.0 g hardener I) as precursors of the embedding resin, and samples were gradually transferred from 100% ethanol to increasing concentrations of the resin substitution solutions [51,52]. During each substitution step, samples were vacuum-infiltrated under reduced pressure and then incubated on a rotator for at least 12 h [51]. For embedding, the resin solution and hardener II were mixed at a 11:1 ratio to prepare the embedding resin [51]. Samples were placed in silicone molds and filled with the resin [51]. After adjusting the orientation of the samples, polymerization was allowed to proceed at 4 °C in the dark for at least 8 h, followed by incubation at room temperature (25 °C) until the resin was completely hardened [51]. The polymerized resin blocks were then glued onto wooden blocks for sectioning [51].

Transverse sections (9–12 μm thick) near the equatorial region were prepared using a rotary microtome (No.1115, Yamato Kohki Industrial Co., Ltd., Saitama, Japan) equipped with a disposable microtome blade (Feather, S35; Feather Safety Razor Co., Ltd., Osaka, Japan). Sections were mounted on slides using a stretching device, stained with a 5.0 mg/L toluidine blue solution (Waldeck GmbH & Co. KG, Münster, Germany), and observed under a digital microscope (DIM-03; Alfa Mirage Co., Ltd., Osaka, Japan). These procedures were adapted from previously published methods (Ishikawa et al. [51]; Kuroiwa [52]).

Cell density was estimated based on the length corresponding to 10 cells in the inner side or 5 cells in the outer side of the mesocarp. For cell size analysis, radial (longitudinal) and circumferential (transverse) diameters of randomly selected cells in the inner and outer regions were measured to calculate the aspect ratio. The radial axis was defined as the longitudinal diameter, and the circumferential axis as the transverse diameter on the equatorial section. Mesocarp cell size was measured separately in the inner- and outer wall regions.

### 3.5. Analysis of Cell Wall Components

Alcohol-insoluble residue (AIR) was prepared from 100 to 300 mg of frozen berry tissue by adding 1.5 mL of 80% ethanol, centrifuging, removing the supernatant, and drying the pellet. The AIR was extracted with 1.6 mL distilled water for 20 h with shaking to obtain the water-soluble fraction. The residue was then extracted sequentially with 1.6 mL of 0.05 M ethylenediaminetetraacetic acid for 20 h (EDTA-soluble fraction) and with 1.6 mL of 0.05 M Na_2_CO_3_ for 20 h (Na_2_CO_3_-soluble fraction).

To remove starch, 1.6 mL sodium acetate buffer (20 mM, pH 6.5) was added to the residue, followed by incubation in boiling water for 5 min. After cooling to room temperature, 320 μL of a mixed enzyme solution containing 8 units mL^−1^ α-amylase (Sigma-Aldrich Japan, Tokyo, Japan) and 8 units mL^−1^ glucoamylase (Sigma-Aldrich Japan, Tokyo, Japan) was added, and the mixture was incubated at 37 °C for 24 h. After removing the supernatant, the residue was washed twice with distilled water.

The residue was then extracted with 1.6 mL of 4 M KOH for 20 h with shaking to obtain the KOH-soluble fraction (KOHSF). The remaining residue was washed twice with 800 μL of 0.1 N acetic acid and twice with 800 μL of a 1:1 (*v*/*v*) ethanol:diethyl ether mixture. The washed residue was dried at 100 °C for 48 h and used as the insoluble material.

As pectin-related components, uronic acid contents of the H_2_O-, EDTA-, and Na_2_CO_3_-soluble fractions were quantified using the m-hydroxydiphenyl method [53]. Total sugar contents of all fractions were determined by the phenol-sulfuric acid method [54]. For cellulose-related sugars, the insoluble material was dissolved in 0.2 mL of 72% sulfuric acid and incubated for 12 h at room temperature. The solution was then diluted with 2.8 mL of distilled water and analyzed using the phenol–sulfuric acid method. Absorbance was measured at 520 nm for uronic acids (as galacturonic acid equivalents) and at 490 nm for total sugars (as glucose equivalents). All cell wall component contents were expressed on a 100 g fresh weight basis. These methods followed those of Oida et al. [5,8] with minor modifications.

### 3.6. RNA Isolation

All samples were pulverized under frozen conditions prior to RNA extraction. Total RNA was extracted using the Cica genius^®^ RNA Prep Kit (for Plant) (Kanto Chemical Co., Inc., Tokyo, Japan). The modified extraction buffer described by McQuinn et al. [55] was used.

### 3.7. RNA Sequencing and Expression Analysis

RNA libraries were prepared using the NEBNext^®^ Ultra™ II Directional RNA Library Prep Kit for Illumina^®^ (New England Biolabs, Inc., Ipswich, MA, USA). Paired-end sequencing (150 bp) was performed on a DNBSEQ-Q7 platform (MGI). Raw reads were assessed using FastQC and trimmed with Trimmomatic v0.39 (Table S1). Trimmed reads were mapped to the ‘Shine Muscat’ genome available in PLANT GARDEN using HISAT2 v2.2.1 [56]. Read counts and TPM values were calculated with StringTie v2.2.3. Annotation of differentially expressed genes (DEGs) was obtained by local BLAST (version 2.16.0) searches against *A. thaliana* against *A. thaliana* reference sequences (TAIR10.1), with a significance threshold of E < 1 × 10^−5^.

### 3.8. Statistical Analysis

Differences between means in the fracture tests and in the analyses of plant cell wall components were evaluated using Welch’s *t*-test. For histological analyses, Welch’s *t*-test was used for all comparisons of means except for the comparison of mesocarp cell size. For the comparison of mesocarp cell size in the histological analysis, an *F*-test (two-sample for variances) was applied.

The baseMan, log_2_ fold change (log_2_FC), and *padj* (FDR-adjusted *p*-value) were calculated using DESeq2 in R v4.4.1. PCA was performed using values normalized for library size differences (total read counts) by DESeq2 via size factors and transformed by variance stabilizing transformation (VST). DEGs were defined as genes with *padj* < 0.05 based on read count data.

Similarity in gene expression patterns was assessed in R using TPM values transformed as log_2_ (TPM + 1). Pearson correlation coefficients with the gene of interest (*VSMuph2.0_chr11.g0320*) were computed using the base R function cor(). Genes with correlation coefficients of *r* ≥ 0.8 were extracted as similar genes. Genes with TPM = 0 across all samples were excluded from the analysis.

## 4. Conclusions

GA-based seedless cultivation in ‘Shine Muscat’ was associated with a clear shift in intact-berry fracture behavior at harvest. GA-treated berries exhibited a higher fracture load and a lower fracture strain than non-treated berries at DAFB105, indicating greater resistance to first failure with reduced deformation capacity, while toughness did not differ significantly. Pronounced differences in pericarp/mesocarp histology accompanied this mechanical shift. GA-treated berries had a thicker pericarp at both DAFB39 and DAFB105 and showed lower mesocarp cell density, together with greater cell-size heterogeneity and enhanced radial cell expansion. These features provide a plausible tissue-level basis for altered fracture-type texture, because both cellular architecture and cell-to-cell adhesion contribute to failure modes in fleshy fruit tissues. Cell wall fractionation further indicated stage-dependent changes in pectin-enriched pools. Uronic acid contents in water-, EDTA-, and Na_2_CO_3_-soluble fractions were lower in GA-treated berries at specific stages, whereas hemicellulose- and cellulose-related fractions showed no significant quantitative differences under our analytical conditions. Although fraction abundance alone cannot resolve pectin chemistry, these results support the view that GA treatment alters the balance of pectin solubilization/retention in a stage-dependent manner, which could influence middle-lamella mechanics and thus fracture behavior.

Transcriptome profiles were consistent with a developmental transition in candidate processes underlying these phenotypes. During early development (DAFB10–24), GA-responsive expression of putative cell expansion and primary-wall remodeling genes (*EXO/EXP/XTH*) supports a scenario in which GA promotes primary-wall extensibility and radial mesocarp expansion. At maturity (DAFB105), non-treated berries exhibited higher expression of *ACO1-like* and *ERF-like* transcripts together with stress-/senescence-associated candidates (including *NAC87-like* and *wound-responsive* genes) and pectin-modifying enzyme genes (*PME-* and *PG/PL-like*), suggesting relatively enhanced ethylene-/senescence-associated transcriptional programs that may facilitate pectin remodeling and contribute to a softer and/or gummy fracture behavior.

Collectively, our integrative approach links intact-berry fracture behavior to stage-resolved changes in mesocarp cellular architecture and pectin-centered wall traits under GA-based seedless cultivation. The anatomical descriptors and pectin-related soluble fractions highlighted here provide candidate intermediate traits for cultivar comparison and management optimization. Future studies that couple tissue-resolved mechanical analyses (skin vs. adjacent flesh) with detailed pectin chemistry (degree/pattern of methylesterification, Ca^2+^ association, and polymer depolymerization) will be essential to validate the mechanistic roles of the wall-remodeling and cell-adhesion candidates suggested by our transcriptome profiles.

## Figures and Tables

**Table 1:** Effects of GA treatment on berry texture traits in ‘Shine Muscat’.

	Fracture Load (N)	Fracture Strain (%)	Toughness (kJ/m^3^)	Diameter (mm)
GA-treated (*n* = 8)	7.21 ± 0.14 ^z,^*	18.8 ± 0.6	106.8 ± 4.6	26.5 ± 0.2 *
Non-treated (*n* = 4)	6.64 ± 0.08	25.0 ± 0.7 **	119.7 ± 3.8	25.3 ± 0.3

^z^ The data are means ± SE. * *p* < 0.05, ** *p* < 0.01, Welch’s *t*-test.

## Data Availability

The RNA-Seq data have been submitted to the DDBJ Sequence Read Archive (DRA) under BioProject accession PRJDB39879 (Run: DRR892648-DRR892665).

## Supplementary Materials

The following supporting information can be downloaded at: https://www.mdpi.com/article/10.3390/plants15020287/s1, Table S1. RNA-Seq read data quality summary; Table S2. DEGs DAFB10; Table S3. DEGs DAFB24; Table S4. DEGs DAFB105; Table S5. High correlation genes with ACO1 like; Figure S1. Cell size variability in the mesocarp; Figure S2. TPM values of *VSMuph2.0_chr102g0031*(ACC synthase 1).
