# Supplementary material for: Gibberellin-Treated Seedless Cultivation Alters Berry Fracture Behavior, Cell Size and Cell Wall Components in the Interspecific Hybrid Table Grape (Vitis labruscana × Vitis vinifera) ‘Shine Muscat’
Source: Plants (Basel). 2026 Jan 17;15(2):287. doi: 10.3390/plants15020287 (PMC12845008; doi:10.3390/plants15020287)
Supplement: Supplementary file 1 [file plants-15-00287-s001.zip › SupplementaryFigures.pptx]

## Slide 1
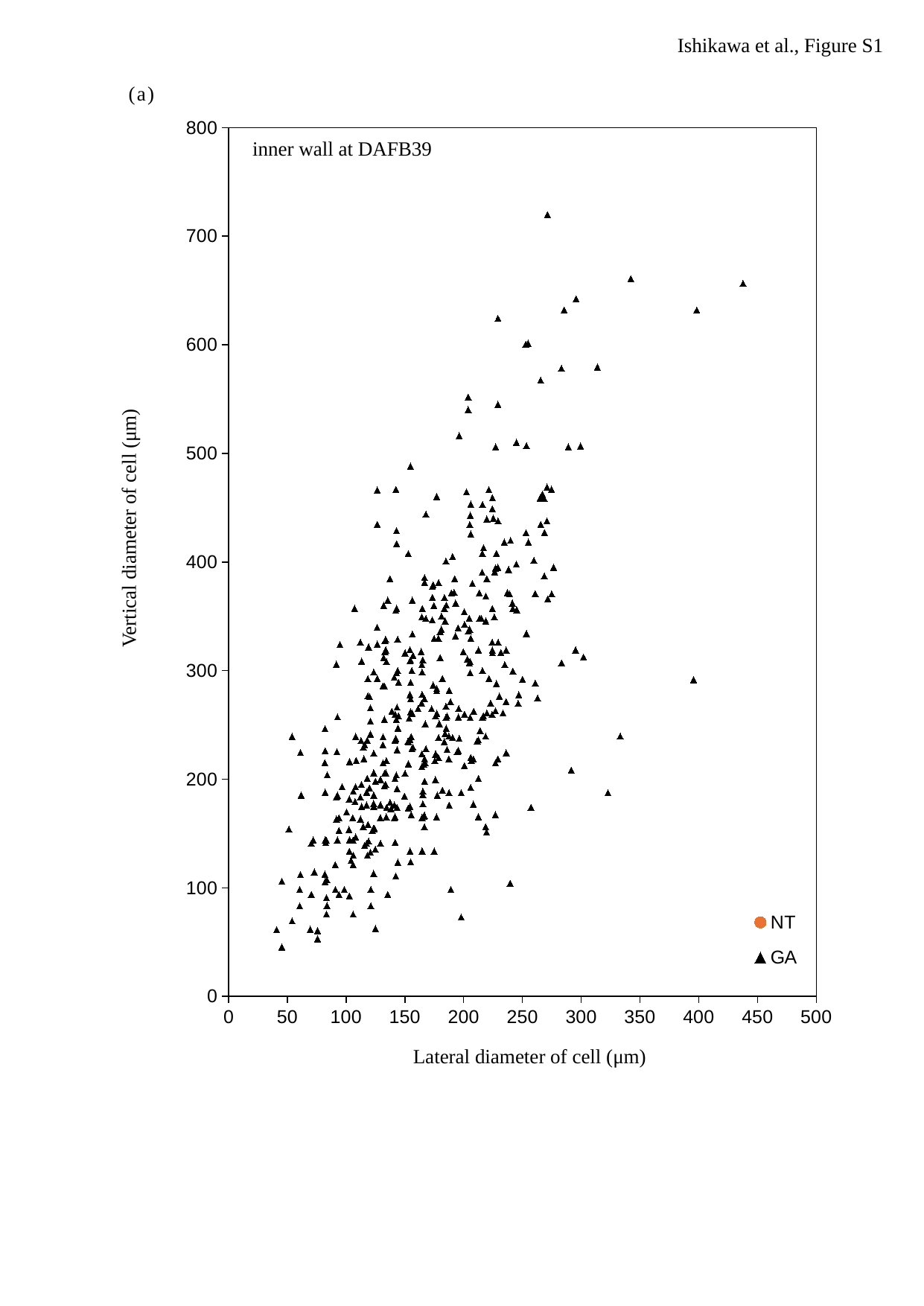

Ishikawa et al., Figure S1
(a)
### Chart
| Category | | |
|---|---|---|inner wall at DAFB39
Vertical diameter of cell (μm)
Lateral diameter of cell (μm)

## Slide 2
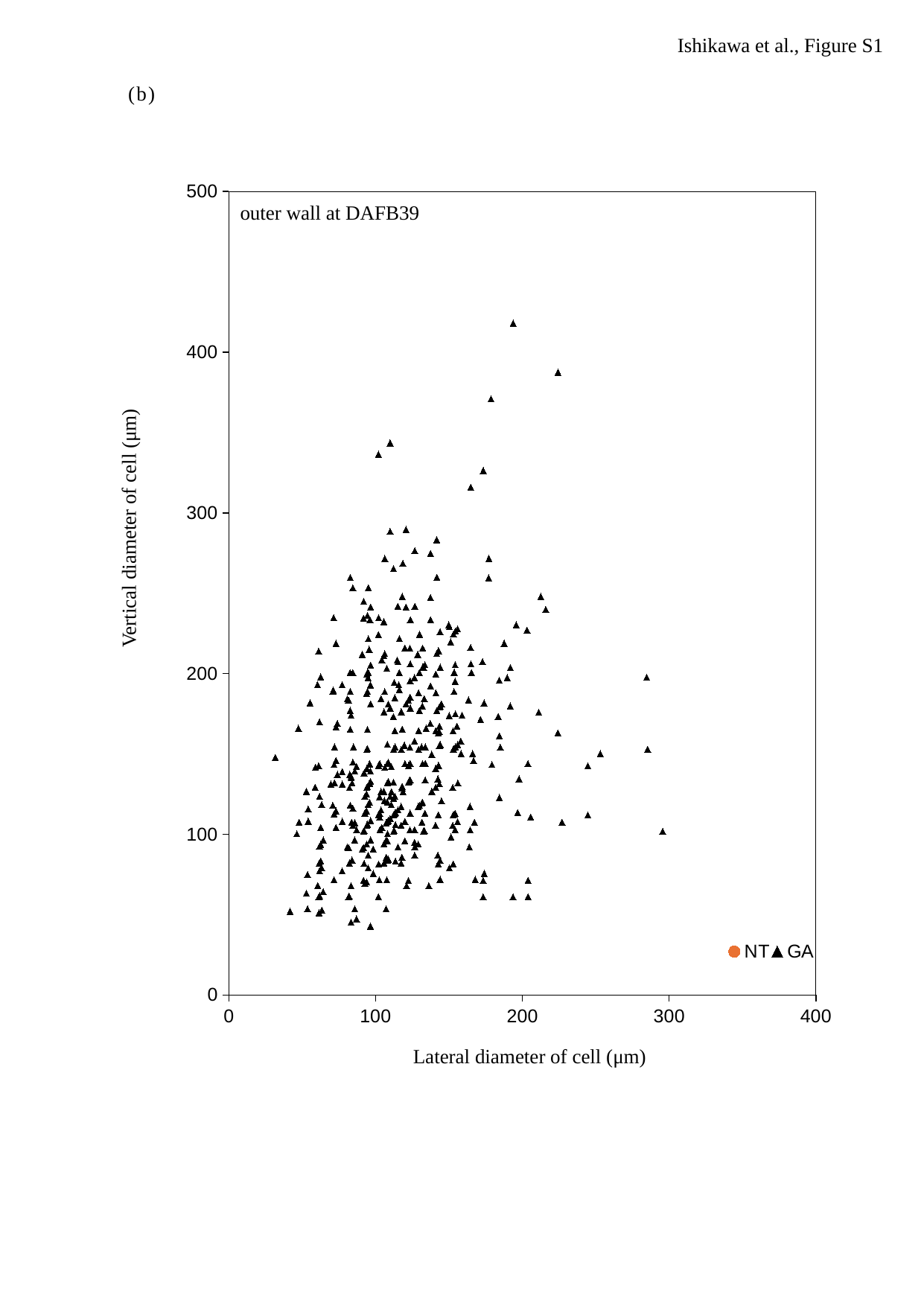

Ishikawa et al., Figure S1
(b)
### Chart
| Category | | |
|---|---|---|outer wall at DAFB39
Vertical diameter of cell (μm)
Lateral diameter of cell (μm)

## Slide 3
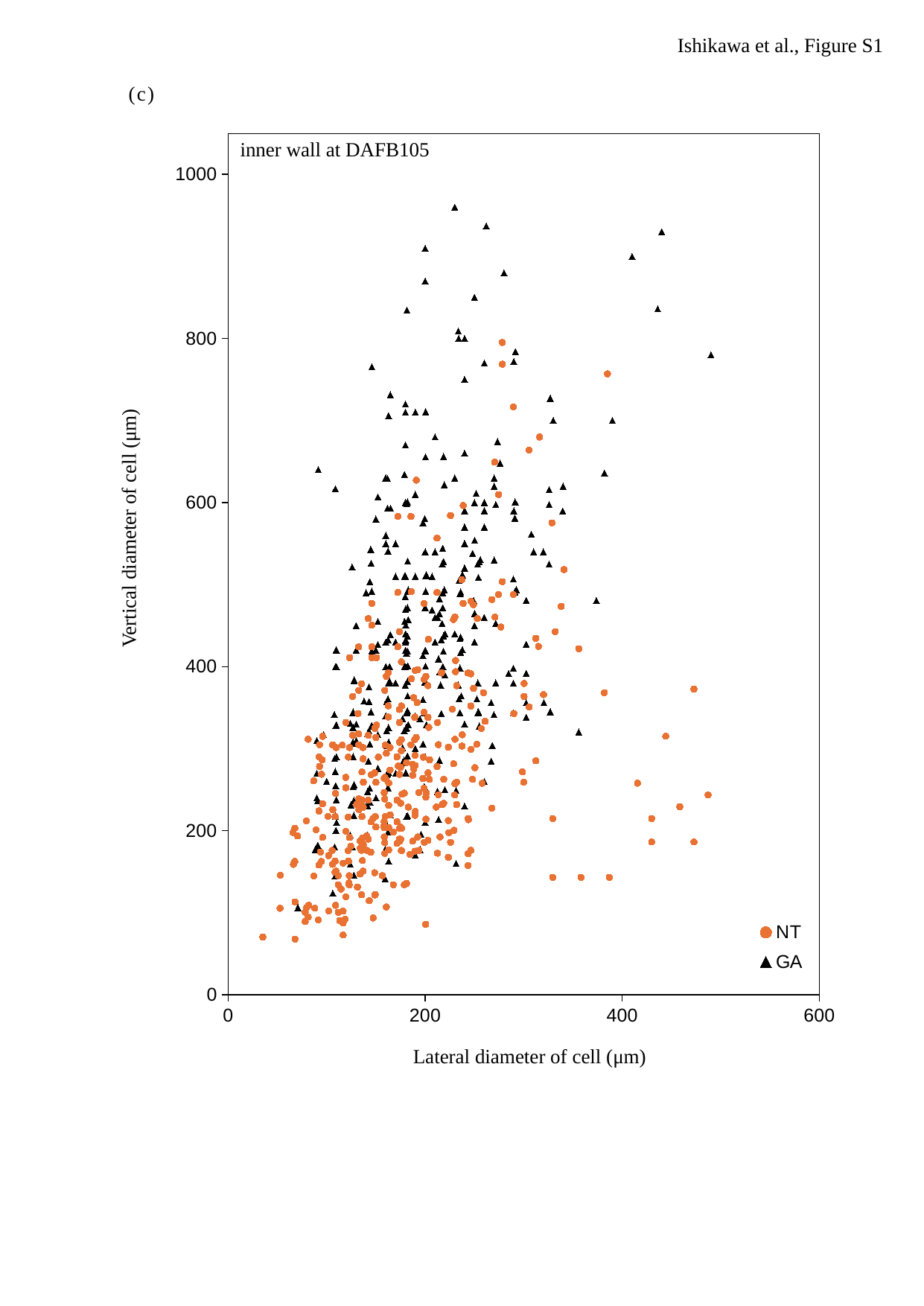

Ishikawa et al., Figure S1
(c)
### Chart
| Category | | |
|---|---|---|inner wall at DAFB105
Vertical diameter of cell (μm)
Lateral diameter of cell (μm)

## Slide 4
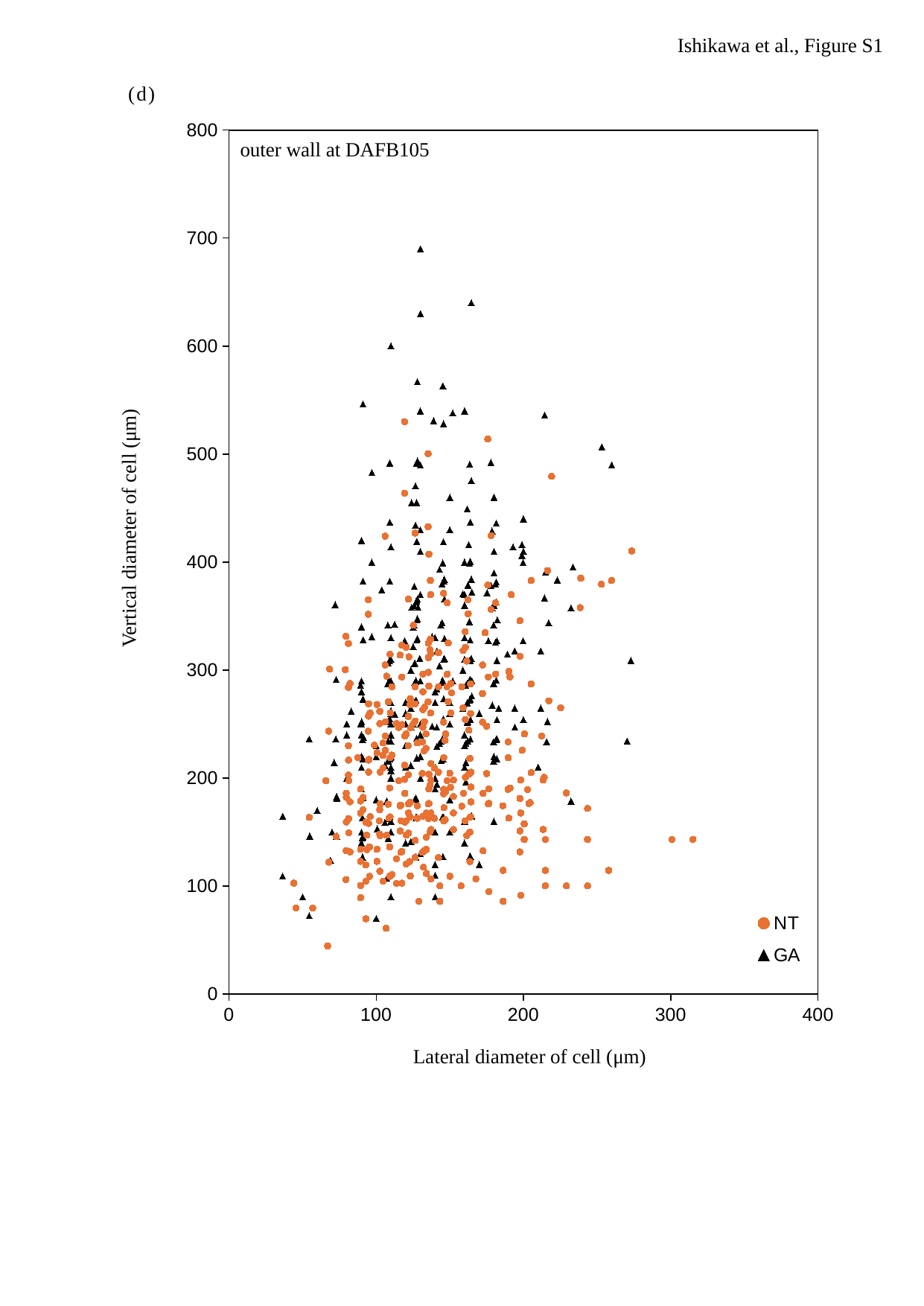

Ishikawa et al., Figure S1
(d)
### Chart
| Category | | |
|---|---|---|outer wall at DAFB105
Vertical diameter of cell (μm)
Lateral diameter of cell (μm)

## Slide 5
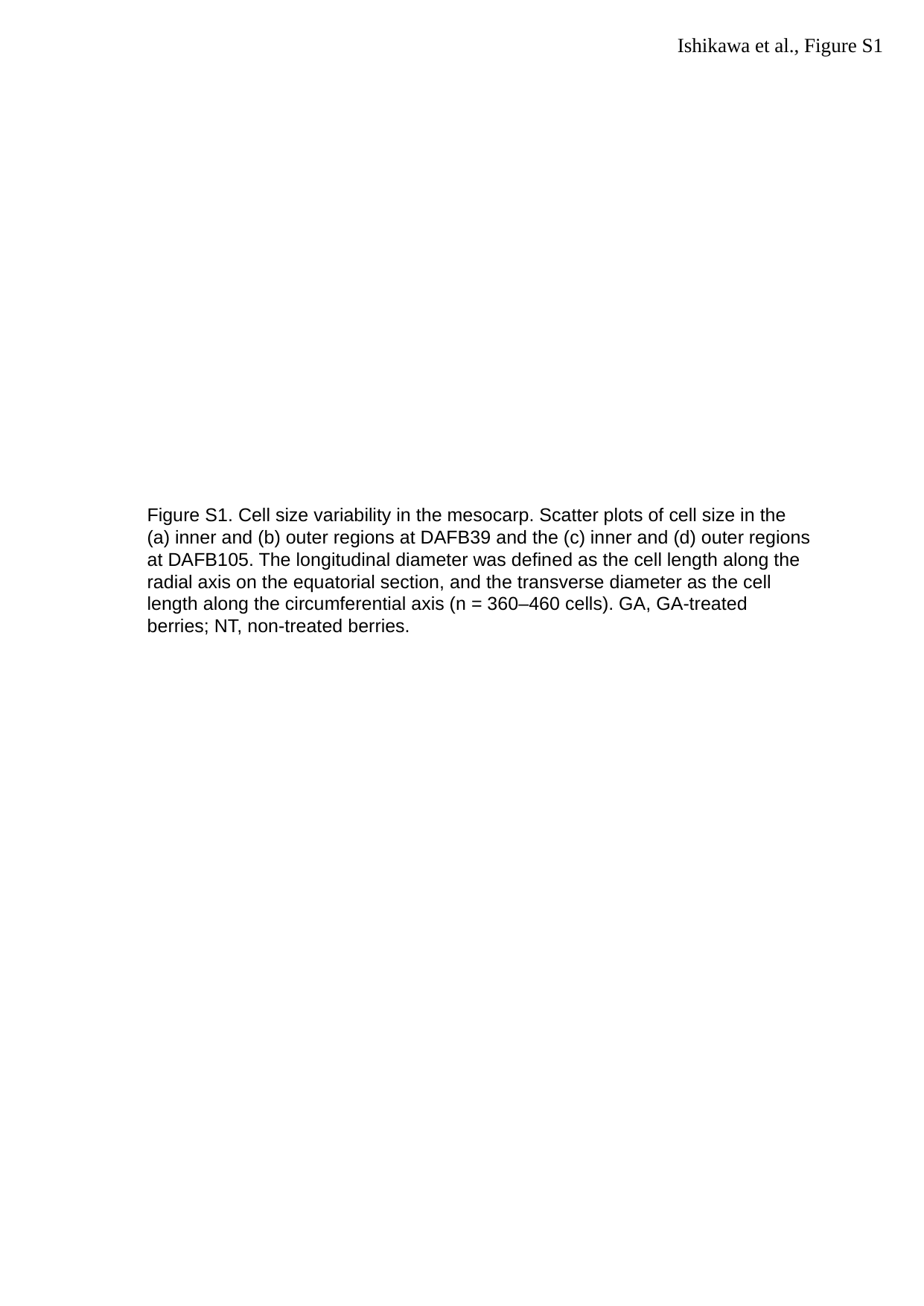

Ishikawa et al., Figure S1
Figure S1. Cell size variability in the mesocarp. Scatter plots of cell size in the (a) inner and (b) outer regions at DAFB39 and the (c) inner and (d) outer regions at DAFB105. The longitudinal diameter was defined as the cell length along the radial axis on the equatorial section, and the transverse diameter as the cell length along the circumferential axis (n = 360–460 cells). GA, GA-treated berries; NT, non-treated berries.

## Slide 6
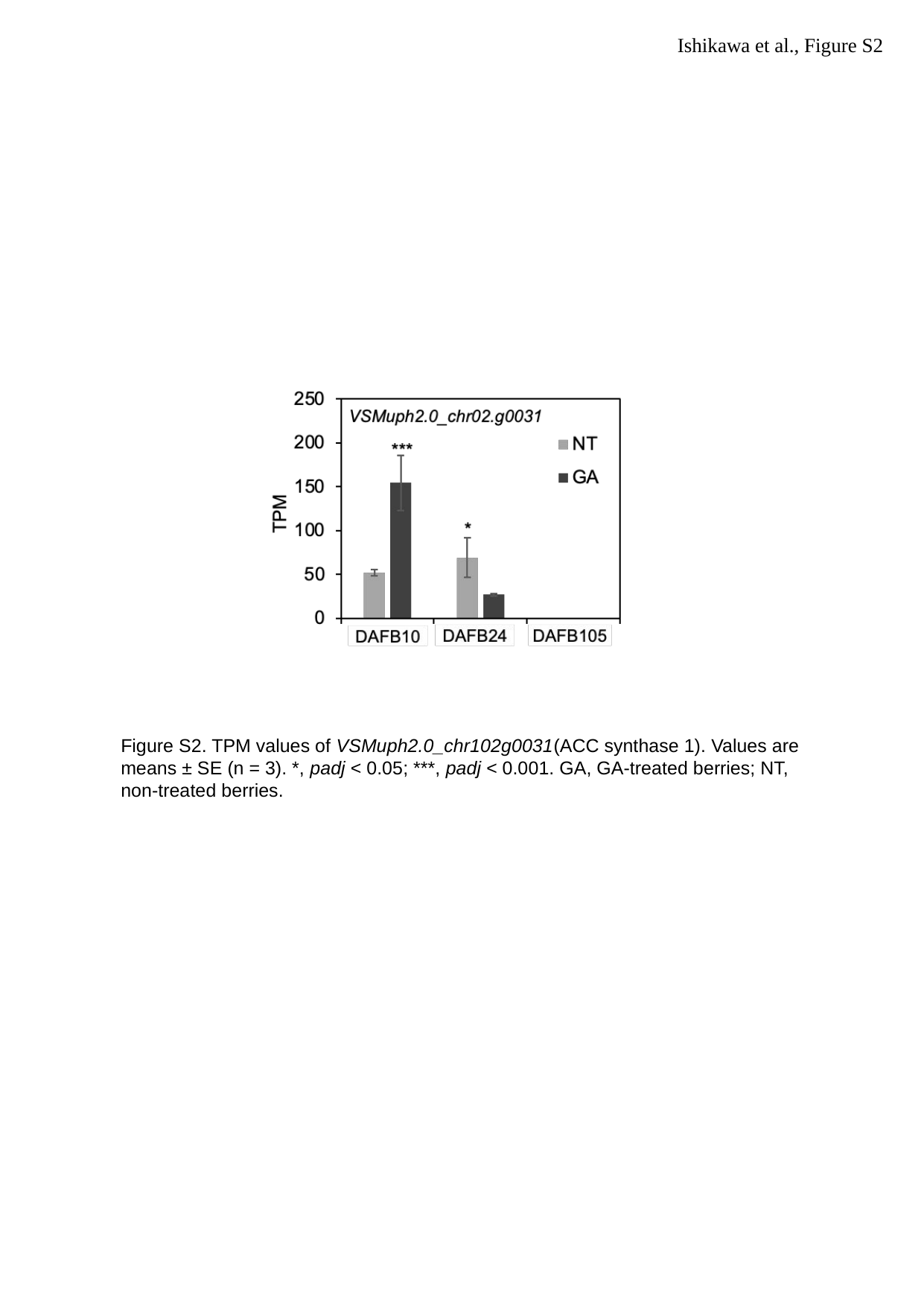

Ishikawa et al., Figure S2
Figure S2. TPM values of VSMuph2.0_chr102g0031(ACC synthase 1). Values are means ± SE (n = 3). *, padj < 0.05; ***, padj < 0.001. GA, GA-treated berries; NT, non-treated berries.
